# GEOGRAPHIC AVAILABILITY OF SUBSIDIZED SENIOR HOUSING AND NEIGHBORHOOD DISPARITIES IN MISSOURI

**DOI:** 10.1093/geroni/igac059.2771

**Published:** 2022-12-20

**Authors:** Byeongju Ryu, Jihye Baek, Sojung Park, Yung Chun, Takashi Amano, BoRin Kim

**Affiliations:** Washington University in St. Louis, St. Louis, Missouri, United States; Washington University in St. Louis, St. Louis, Missouri, United States; Washington University in Saint Louis, Saint Louis, Missouri, United States; Washington University in St. Louis, St. Louis, Missouri, United States; Rutgers University-Newark, Newark, New Jersey, United States; University of New Hampshire, Durham, New Hampshire, United States

## Abstract

Subsidized senior housing (SSH) provides an affordable option to low-income older adults for economic stability and aging-in-place. To date, limited attention has been given to the geographic availability of SSH and its relation to neighborhood characteristics (e.g., socioeconomic characteristics and availability of supportive services). The current study aims to describe (1) the availability of government-subsidized senior housing (SSH-availability) in Missouri and examine (2) to what extent the housing availability is associated with county-level neighborhood characteristics. First, SSH-availability in each county was measured with the number of subsidized units for older adults divided by the eligible population (i.e., aged 65 or over, income below poverty level, and spending 30% or more of income on housing). The Geographic Information System (GIS) was used to examine the distribution of SSH in Missouri. Second, based on existing literature, we used principal component analysis with multiple indicators to create two neighborhood characteristics (i.e., socioeconomic deprivation and health and social service indices). Non-parametric bivariate analyses (Kruskal-Wallis tests) were conducted with the availability quartiles and each index. Our findings showed that in 66 counties (57% of entire counties in Missouri), SSH-availability was below 20%. Bivariate analyses showed SSH-availability was significantly associated with both neighborhood characteristics: counties with higher SSH-availability were more likely to be socioeconomically deprived and face a lack of health and social services. The current study suggests the government should address the geographic disparities in SSH for low-income older adults. Also, more supportive services are needed around SSH to facilitate aging-in-place of vulnerable older adults.

